# Re-establishment of the epigenetic state and rescue of kinome deregulation in Ts65Dn mice upon treatment with green tea extract and environmental enrichment

**DOI:** 10.1038/s41598-020-72625-z

**Published:** 2020-09-29

**Authors:** I. De Toma, M. Ortega, S. Catuara-Solarz, C. Sierra, E. Sabidó, M. Dierssen

**Affiliations:** 1 Centre for Genomic Regulation (CRG), The Barcelona Institute of Science and Technology, Dr. Aiguader 88, 08003 Barcelona, Spain; 2 Universitat Pompeu Fabra (UPF), Dr. Aiguader 88, 08003 Barcelona, Spain; 3 Proteomics Unit, Centre for Genomic Regulation (CRG), The Barcelona Institute of Science and Technology, Dr. Aiguader 88, 08003 Barcelona, Spain; 4 Centro de Investigación Biomédica en Red de Enfermedades Raras (CIBERER), Barcelona, Spain

**Keywords:** Developmental disorders, Diseases of the nervous system, Proteomics

## Abstract

Down syndrome (DS) is the main genetic cause of intellectual disability due to triplication of human chromosome 21 (HSA21). Although there is no treatment for intellectual disability, environmental enrichment (EE) and the administration of green tea extracts containing epigallocatechin-3-gallate (EGCG) improve cognition in mouse models and individuals with DS. Using proteome, and phosphoproteome analysis in the hippocampi of a DS mouse model (Ts65Dn), we investigated the possible mechanisms underlying the effects of green tea extracts, EE and their combination. Our results revealed disturbances in cognitive-related (synaptic proteins, neuronal projection, neuron development, microtubule), GTPase/kinase activity and chromatin proteins. Green tea extracts, EE, and their combination restored more than 70% of the phosphoprotein deregulation in Ts65Dn, and induced possible compensatory effects. Our downstream analyses indicate that re-establishment of a proper epigenetic state and rescue of the kinome deregulation may contribute to the cognitive rescue induced by green tea extracts.

## Introduction

Down Syndrome (DS) is the most common genetic cause of intellectual disability and is due to the presence of three copies of chromosome 21 (HSA21). However, DS cognitive impairment is still not amenable to therapy. The most important reason is that the mechanism(s) of the cognitive impairment are still not well understood. Recent evidence has shown that green tea extracts containing epigallocatechin-3-gallate (EGCG), a green tea flavonol, improve the cognitive phenotype in trisomic Ts65Dn mice, as well as in individuals with DS^1,2^, providing a unique opportunity to study the molecular mechanisms underlying its beneficial effects. EGCG is known for its antioxidant properties, that could counteract the oxidative stress caused by the upregulation of CuZnSOD1^3^ in DS. However, EGCG has many other properties that could also be beneficial in DS. It directly regulates and interacts with proteins involved in the cell membrane integrity, signal transduction, transcription factors, DNA methylation, phosphorylation, mitochondrial function and autophagy^4^. Of interest for DS, EGCG inhibits the Dual Specificity Tyrosine-Phosphorylation-Regulated Kinase 1A (DYRK1A), one of the most important genes in the pathogenesis of DS^5,6^. Previous work from our group showed that EGCG partially rescues the effects of overexpression of *DYRK1A* on the hippocampal proteome and phosphoproteome of TgDyrk1A mice^7^. However, the extent to which all these mechanisms apply to a trisomy scenario is unknown.

Interestingly, EGCG shares important similarities with environmental enrichment (EE). In Ts65Dn mice, EE improved spatial learning and memory^8^ , visual function^9^, normalized brain inhibition, enhanced hippocampal synaptic plasticity, increased branching in pyramidal cells^10^ and rescued postnatal neurogenesis defect^11^.

Moreover, we recently found that the combination of EE plus EGCG treatment is more efficient than EE or EGCG alone to ameliorate age-associated cognitive impairment of old Ts65Dn mice^12^ and it improves corticohippocampal-dependent learning and memory deficits in young trisomic mice. Those effect are possibly the consequences of the restoration of CA1 hippocampal dendritic spine density, and mitigation of the disturbed excitation/inhibition synaptic puncta imbalance^13^.

To get insight into these mechanisms we analyzed changes in protein abundances and phosphorylation in Ts65Dn mice, and their disomic counterparts in baseline conditions and upon three treatments known to improve cognition in Ts65Dn: (i) green tea extract containing EGCG, (ii) environmental enrichment (EE), and (iii) their combination.

## Results

### Ts65Dn hippocampal (phospho-)proteome

Using quantitative mass spectrometry-based proteomics (LC–MS/MS) on whole proteome extracts from Ts65Dn and WT mice, treated or not with EGCG containing green tea extracts (“green tea”), EE, or green tea extracts + EE (n total = 40 mice), we identified 2633 proteins, and 4705 phosphopeptides belonging to 1759 phosphorylated proteins. We fitted the data into a linear model to evaluate the changes related to the genotype, to the treatments, and their interaction, setting a threshold of adjusted p-value and fold change of p < 0.05, and |log_2_FC| > 0.3.

### Protein dysregulated in Ts65Dn are not limited to the triplicated HSA21 genes

**Figure 1:**
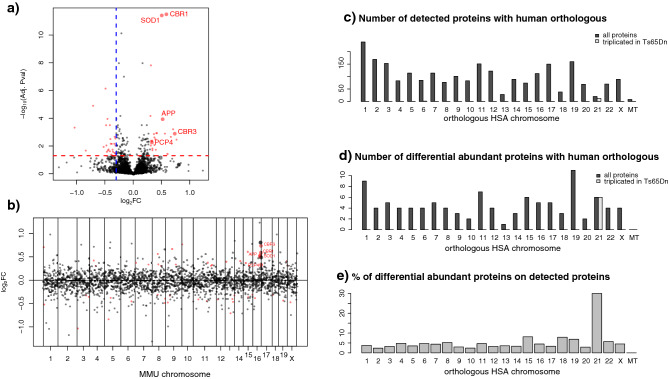
Differentially abundant proteins in each orthologous human chromosome. (**a**) Log2 fold change of the TS.NT − WT.NT and significance (− log10 of the adjusted p-value) of all evaluated proteins. Significant proteins are indicated in red, proteins from the trisomic regions are visualized in bigger size. The dashed horizontal red line indicates the threshold of adjusted p-value (0.05), the dashed blue vertical lines the threshold of log2FC (0.3). The significantly upregulated triplicated genes are labelled. (**b**) Distribution of the detected proteins along the murine chromosomes, with the y-axis showing the protein log2 fold changes. Dot colours, sizes, and labels are the same as in (**a**). (**c**) Distribution of all detected proteins with a human orthologous across the human chromosomes. (**d**) Distribution of the proteins whose levels are changing in trisomic mice across the human chromosomes. (**e**) Percentage (%) of differentially abundant proteins over the total proteins detected orthologous to each human chromosome.

We found a significant increase in the abundance of 48 proteins (red dots in Fig. 1A,B), 20 of which were low or absent in WT hippocampi, and significantly decreased abundance of 67 proteins (Fig. 1A,B, Table 1).

**Table 1:** Summary statistics for the proteomic study.

	Detected proteins (n)	Up-regulated (n)	Present in TS, low/absent in WT	Down-regulated (n)	Low/absent in TS, present in WT
TS.NT − WT.NT	2405	28	20	31	36
WT.greentea − WT.NT	2456	29	24	41	14
WT.EE − WT.NT	2449	24	38	37	26
WT.greentea + EE − WT.NT	2450	48	29	44	19
TS.greentea − TS.NT	2414	33	38	28	13
TS.EE − TS.NT	2396	53	35	47	27
TS.greentea + EE − TS.NT	2395	46	39	30	23
Rescued by the green tea extract	36	6	3	13	14
Rescued by EE	37	11	1	14	11
Rescued by the combined treatment	44	9	5	13	17

Proteins were considered differentially abundant if they had an adjusted p-value lower than 0.05 and a log2 (Fold Change) greater than 0.3 or lower than − 0.3.

TS, ts65Dn mice. WT, wild type mice. NT, not treated. greentea, “EGCG-containing green tea extract”. EE, Environmental Enrichment.

Mapping all the detected protein to their respective human orthologous genes (Fig. 1C–E), revealed that their distribution across all chromosomes was as expected given the gene density of each chromosome. Since HSA21 is a gene-poor chromosome, only 19 HSA21 orthologous genes had a protein detected in our dataset. Of these, 12 are encoded by genes triplicated in Ts65Dn, of which 6 were found differentially expressed, with 5 upregulated (Table 2). When calculating the ratio of differentially abundant proteins over detected proteins, HSA21 was the one with the highest percentage (over 30%) (Fig. 1E). In Ts65Dn there are also 64 genes non-orthologous to HSA21 (mm10 release), but only the one coding for the ezrin protein (whose orthologous encoding gene is on HSA6) was detected in our proteomic analysis, and it was not differentially abundant when comparing trisomic versus wild type mice.

**Table 2:** Summary statistics for detected proteins with orthologous encoded by HSA21.

UNIPROT	3 copies in Ts65Dn	MGI symbol	Description	log_2_FC	Statistically significant
Q62426	No	Cstb	Cystatin B	− 0.23	No
P12382	No	Pfkl	Phosphofructokinase, liver, B-type	− 0.2	No
O35136	No	Ncam2	Neural cell adhesion molecule 2	− 0.16	No
P97450	Yes	Atp5j	ATP synthase, H + transporting, mitochondrial F0 complex, subunit F	0.09	No
P12023	Yes	App	Amyloid beta (A4) precursor protein	0.52	Yes
Q8K354	Yes	Cbr3	Carbonyl reductase 3	0.74	Yes
Q8BHB9	Yes	Clic6	Chloride intracellular channel 6	NA	No
Q9DB20	Yes	Atp5o	ATP synthase, H + transporting, mitochondrial F1 complex, O subunit	0.01	No
Q9Z0R4	Yes	Itsn1	Intersectin 1 (SH3 domain protein 1A)	0.26	No
Q64737	Yes	Gart	Phosphoribosylglycinamide formyltransferase	0.8	No
P08228	Yes	Sod1	Superoxide dismutase 1, soluble	0.51	Yes
Q8BK30	No	Ndufv3	NADH dehydrogenase (ubiquinone) flavoprotein 3	− 0.13	No
P42932	Yes	Cct8	Chaperonin containing Tcp1, subunit 8 (theta)	0.01	No
Q8K183	No	Pdxk	Pyridoxal (pyridoxine, vitamin B6) kinase	− 0.09	No
P50114	No	S100b	S100 protein, beta polypeptide, neural	− 0.05	No
P58064	Yes	Mrps6	Mitochondrial ribosomal protein S6	Absent	Yes
P48758	Yes	Cbr1	Carbonyl reductase 1	0.59	Yes
Q9D172	No	D10Jhu81e	DNA segment, Chr 10, Johns Hopkins University 81 expressed	0.19	No
Q921W4	Yes	Cryzl1	Crystallin, zeta (quinone reductase)-like 1	0.81	No
P63054	Yes	Pcp4	Purkinje cell protein 4	0.33	Yes

Uniprot ID, the MGI symbols, and log_**2**_FC of the TS.NT − WT.NT contrast showing if a protein is statistically significant or not, and if the gene is triplicated in Ts65Dn.

### Kinome deregulation in Ts65Dn

We found 576 phosphopeptides—corresponding to 442 proteins—that exhibited increased abundances in Ts65Dn compared to their WT counterparts, whereas 301 phosphopeptides, mapping to 250 different proteins, were decreased in abundance (Table 3).

**Table 3:** Summary statistics for the phospho-proteomic study.

	Detected phospho-peptides (n proteins)	Up-phosphorylated peptides (n)	Phospho-peptides present in TS, low/absent in WT (n)	Down-phosphorylated peptides (n)	Phospho-peptides low/absent in TS, present in WT (n)
TS.NT − WT.NT	3020 (1352)	37 (37)	539 (405)	53 (50)	248 (200)
WT.greentea − WT.NT	3074 (1396)	39 (39)	591 (547)	37 (34)	198 (160)
WT.EE − WT.NT	3185 (1402)	54 (51)	701 (512)	48 (44)	211 (173)
WT.greentea + EE − WT.NT	2557 (1198)	69 (60)	339 (260)	43 (37)	182 (157)
TS.greentea − TS.NT	4149 (1652)	56 (53)	747 (546)	60 (57)	677 (484)
TS.EE − TS.NT	3495 (1479)	55 (54)	467 (363)	29 (28)	362 (302)
TS.greentea + EE − TS.NT	3587 (1531)	32 (32)	557 (411)	28 (27)	296 (248)
Rescued by the green tea extract	471 (371)	19 (19)	282 (235)	22 (22)	148 (133)
Rescued by EE	362 (295)	16 (16)	195 (170)	21 (21)	130 (119)
Rescued by the combined treatment	348 (276)	11 (11)	182 (157)	20 (20)	136 (116)

Number of phospho-peptides and the corresponding number of proteins (in brackets) changing in the different experimental conditions. Phospho-peptides were considered differentially expressed if having an adjusted p-value lower than 0.05 and a log2 (Fold Change) greater than 0.3 or lower than − 0.3. The proteins containing at least one differentially expressed phosphopeptide were called “differentially phosphorylated”.

TS, ts65Dn mice. WT, wild type mice. NT, not treated. greentea, “EGCG-containing green tea extract”. EE, Environmental Enrichment.

We aligned all the phosphopeptides with higher abundance (or exclusively present) in Ts65Dn mice creating a sequence logo of the trisomic phosphorylation motif (Supplementary Fig. S1). This showed that 502 out of 576 phosphopeptides had a phosphorylated Serine, with 176 of these also having a proline in position + 1 and 43 a proline in position − 2. The proteins changing their phosphorylation were different from those changing in abundance, with only a 10% of the dysregulated proteins changing both their levels and phosphorylation.

When analyzing the kinases with abundance or phosphorylation levels significantly changing in trisomic mice, we found 45 deregulated kinases. Of those, six kinases showed changes in abundance (2 lower and 4 higher). We also detected 12 kinases with reduced phosphorylation, which we identified mainly in the CAMK (Ca^2+^/calmodulin-dependent protein kinase) family (p < 0.001, Fisher Test); and 20 kinases with increased phosphorylation levels compared to the WT, belonging to the CAMK family (p < 0.001, Fisher Test), the *Tyrosine Kinase*-Like (*TKL*) (p < 0.001, Fisher Test), Ca^2+^/calmodulin-dependent protein kinase (CMGC) (p < 0.001, Fisher Test), protein kinase A, G, and C families (AGC) (p < 0.001, Fisher Test), Cell Kinase 1 (CK1) (p < 0.05, Fisher Test), and "Sterile" serine/threonine kinases (STE) (p < 0.05, Fisher Test) groups. Finally 7 kinases belonging to the CAMK group (p < 0.001, Fisher Test) and the AGC group (p < 0.01, Fisher Test) presented several phosphorylation sites up- or down-phosphorylated (Table 4).

**Table 4:** Summary statistics for deregulated kinases showing altered abunances or phosphorylation state.

MGI symbol	Uniprot	Kinase name	Group	Family	Sub-family	Abundance	Phosphorylation	Rescued by green tea	Rescued by EE	Rescued by green tea + EE
Aak1	Q3UHJ0	AP2-associated protein kinase 1	Other	NAK	NA		UP	X		
Abr	Q5SSL4	Active breakpoint cluster region-related protein	Atypical	BCR	NA		DOWN	X		
Braf	P28028	Serine/threonine-protein kinase B-raf	TKL	RAF	NA		UP	X	X	X
Brsk1	Q5RJI5	Serine/threonine-protein kinase BRSK1	CAMK	CAMKL	BRSK		UP		X	
Brsk2	Q69Z98	Serine/threonine-protein kinase BRSK2	CAMK	CAMKL	BRSK		UP/DOWN	X	X	X
Camk1d	Q8BW96	Calcium/calmodulin-dependent protein kinase type 1D	CAMK	CAMK1	NA		UP	X	X	X
Camk2a	P11798	Calcium/calmodulin-dependent protein kinase type II subunit alpha	CAMK	CAMK2	NA		DOWN	X	X	X
Camk2b	P28652	Calcium/calmodulin-dependent protein kinase type II subunit beta	CAMK	CAMK2	NA		UP/ DOWN	X	X	X
Camkk1	Q8VBY2	Calcium/calmodulin-dependent protein kinase kinase 1	Other	CAMKK	CAMKK-Meta		DOWN		X	
Camkk2	Q8C078	Calcium/calmodulin-dependent protein kinase kinase 2	Other	CAMKK	CAMKK-Meta		DOWN	X		
Camkv	Q3UHL1	CaM kinase-like vesicle-associated protein	CAMK	CAMK-Unique	NA		UP	X		X
Cdc42bpa	Q3UU96	Serine/threonine-protein kinase MRCK alpha	AGC	DMPK	GEK		DOWN		X	X
Cdk11b	P24788	Cyclin-dependent kinase 11B	CMGC	CDK	CDK11		DOWN	X	X	X
Cdk13	Q69ZA1	Cyclin-dependent kinase 13	CMGC	CDK	CRK7		UP	X		X
Cdk14	O35495	Cyclin-dependent kinase 14	CMGC	CDK	PFTAIRE		UP			
Dclk1	Q9JLM8	Serine/threonine-protein kinase DCLK1	CAMK	DCAMKL	NA		DOWN	X		
Irak4	Q8R4K2	Interleukin-1 receptor-associated kinase 4	TKL	IRAK	NA	UP		X		
Kalrn	A2CG49	HCG2039851	CAMK	Trio	NA	UP				
Kit	P05532	Mast/stem cell growth factor receptor Kit	TK	PDGFR	NA	DOWN				
Ksr1	Q61097	Kinase suppressor of Ras 1	TKL	RAF	KSR		UP	X		
Lmtk2	Q3TYD6	Serine/threonine-protein kinase LMTK2	TK	Lmr	NA		UP	X	X	
Map2k2	Q63932	Dual specificity mitogen-activated protein kinase kinase 2	STE	STE7	NA	UP				
Map3k4	O08648	Mitogen-activated protein kinase kinase kinase 4	STE	STE11	NA		UP/ DOWN	X	X	X
Mapk3	Q63844	Mitogen-activated protein kinase 3	CMGC	MAPK	ERK1	UP			X	X
Mark3	Q03141	MAP/microtubule affinity-regulating kinase 3	CAMK	CAMKL	MARK		DOWN	X		
Mark4	Q8CIP4	MAP/microtubule affinity-regulating kinase 4	CAMK	CAMKL	MARK		UP	X	X	X
Mast1	Q9R1L5	Microtubule-associated serine/threonine-protein kinase 1	AGC	MAST	NA		UP			
Ntrk3	Q6VNS1	NT-3 growth factor receptor	TK	Trk	NA		DOWN	X	X	X
Pak4	Q8BTW9	Serine/threonine-protein kinase PAK 4	STE	STE20	PAKB		UP			
Pak7	Q8C015	Serine/threonine-protein kinase PAK 7	STE	STE20	PAKB		DOWN			
Prkacb	P68181	cAMP-dependent protein kinase catalytic subunit beta	AGC	PKA	NA	DOWN			X	
Prkce	P16054	Protein kinase C epsilon type	AGC	PKC	PKCh		UP/DOWN	X	X	X
Prkcg	P63318	Protein kinase C gamma type	AGC	PKC	PKCa		UP/DOWN	X	X	X
Prpf4b	Q61136	Serine/threonine-protein kinase PRP4 homolog	CMGC	DYRK	PRP4		UP		X	
Raf1	Q99N57	RAF proto-oncogene serine/threonine-protein kinase	TKL	RAF	NA		UP			X
Rps6ka2	Q9WUT3	Ribosomal protein S6 kinase alpha-2	CAMK	RSKb	RSKb		UP	X	X	
Rps6ka2	Q9WUT3	Ribosomal protein S6 kinase alpha-2	AGC	RSK	RSKp90		UP	X	X	
Rps6kc1	Q8BLK9	Ribosomal protein S6 kinase delta-1	AGC	RSKL	NA		UP	X	X	
Snrk	Q8VDU5	SNF-related serine/threonine-protein kinase	CAMK	CAMKL	SNRK		DOWN	X		
Stk11	Q9WTK7	Serine/threonine-protein kinase STK11	CAMK	CAMKL	LKB		UP	X		
Stk39	Q9Z1W9	STE20/SPS1-related proline-alanine-rich protein kinase	STE	STE20	FRAY		UP			
Trim28	Q62318	Transcription intermediary factor 1-beta	Atypical	TIF1	NA		UP/DOWN	X	X	X
Trio	Q0KL02	Triple functional domain protein	CAMK	Trio	NA		UP/DOWN		X	X
Ttbk1	Q6PCN3	Tau-tubulin kinase 1	CK1	TTBK	NA		UP			
Wnk3	Q80XP9	Serine/threonine-protein kinase WNK3	Other	WNK	NA		DOWN			

Table showing the MGI symbols, Uniprot ID, kinase information, and information relative to the abundance and phosphorylation of the kinases and their rescue by our treatments.

### The (phospho-)proteomic alterations in trisomic hippocampus are enriched in neuronal and chromatin-related categories

**Figure 2:**
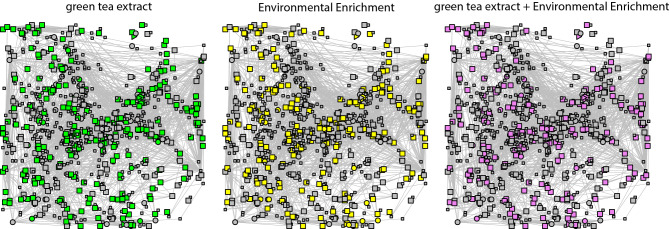
The Ts65Dn’s protein-protein interaction network. In the network the nodes correspond to proteins changing in Ts65Dn mice (*seeds*) and their direct interactors, and edges correspond to known protein–protein interactions. *Seed* proteins are represented as bigger nodes. Circles correspond to proteins changing their abundances, squares to proteins changing their phosphorylation levels, and triangles to proteins changing both their abundance and their phosphorylation levels. The network shows nodes affected by the green tea extract (green), by EE (yellow), or by their combination (purple).

Having identified consistent and reproducible changes in the hippocampal proteome of Ts65Dn, we next interrogated functional associations among differentially expressed proteins. To this aim, we constructed a protein–protein interaction (PPI) network (the *Ts65Dn’s network*) starting from the 694 proteins that showed affected abundance or phosphorylation levels in trisomic mice, and we detected interactions among 381 proteins (*seed* proteins, represented in Fig. 2 as those in bigger size). To this PPI network, we also added the direct interactors ending up with 845 interacting proteins in the extended PPI network (Fig. 2). Both the *seed* proteins and their direct interactors were interacting more than expected by chance, suggesting that the significant changes in abundance observed in our dataset are functionally related (590 observed, versus 188 expected interactions, for *seed* proteins; and 3708 observed compared to 1023 expected, for the extended network). Specifically, we found a total of 1879 interactions between proteins changing their phosphorylation state; 325 interactions between proteins only changing their abundance and 207 interactions between a protein changing abundance and a protein changing phosphorylation levels.

The number of interactions per protein (node degree) in the network followed a log-normal right-skewed distribution. Using this distribution, we detected 22 “hubs” in the network, defined as those appearing in the 5% right tail of the distribution, having at least 42 interactions (Supplementary Fig. S2A,B). Of these hubs, seven were *seed* proteins: RPS27A, SOS1, CREBBP, APP, CTNNB1, DLG4, and PLCG1 (Table 5). Of those, RPS27A, SOS1, CTNNB1, and PLCG1 had many common interactors in the network, whereas APP, CREBBP, and DLG4 had a specific subset of interactors (Supplementary Fig. S2C).

**Table 5:** Hubs of the *Ts65Dn network*.

Interactions (n)	p-value	Symbol	Description	Rescued	Seed	DYRK1A target
92	0.012	Rps27a	Ribosomal protein S27A	Yes	Yes	
78	0.017	Rac1	RAS-related C3 botulinum substrate 1			
73	0.019	Cdc42	Cell division cycle 42			
70	0.021	Rhoa	Ras homolog family member A			
68	0.022	Grb2	Growth factor receptor bound protein 2			Yes
66	0.023	Uba52	Ubiquitin A-52 residue ribosomal protein fusion product 1			
65	0.024	Akt1	Thymoma viral proto-oncogene 1			
58	0.029	Ubc	Ubiquitin C			
57	0.029	Sos1	SON of sevenless homolog 1 (Drosophila)	Yes	Yes	
57	0.029	Trp53	Transformation related protein 53			Yes
56	0.031	Crebbp	CREB binding protein		Yes	
54	0.033	Hras	Harvey rat sarcoma virus oncogene			Yes
52	0.035	Pik3r1	Phosphatidylinositol 3-kinase, regulatory subunit, polypeptide 1 (p85 alpha)			
50	0.037	App	Amyloid beta (A4) precursor protein		Yes	Yes
46	0.043	Ctnnb1	Catenin (cadherin associated protein), beta 1	Yes	Yes	
46	0.043	Dlg4	Discs, large homolog 4 (Drosophila)	Yes	Yes	
46	0.043	Pik3r2	Phosphatidylinositol 3-kinase, regulatory subunit, polypeptide 2 (p85 beta)			
46	0.043	Rac2	RAS-related C3 botulinum substrate 2			
46	0.043	Plcg1	Phospholipase C, gamma 1	Yes	Yes	
43	0.047	Rac3	RAS-related C3 botulinum substrate 3			
42	0.049	Mapk1	Mitogen-activated protein kinase 1			Yes
42	0.049	Shc1	Src homology 2 domain-containing transforming protein C1			

The number of interactions, gene symbol, description and other information is reported. The p-value correspond at the area under the right-skewed distribtution (Supplementary Fig. S1).

The Gene ontology categories found enriched among the deregulated proteins between wild type and trisomic mice belonged to 5 main groups: chromatin-related, neuronal projection/neuron development, microtubule/cytoskeleton, synapsis/cognition, and GTPase/Kinase activity (Supplementary Fig. S3, Table S22).

In an independent cohort of mice we confirmed both the upregulation of the neuronal protein APP, as an important hub protein in the network (Supplementary Fig. S4A,B, 2 × in Ts65Dn mice, p < 0.05) and the deregulation of chromatin-related proteins as shown by the global histone hypo-acetylation in trisomic mice (Supplementary Fig. S4C,D, p < 0.05).

### Chromatin and neuronal related categories are rescued by pro-cognitive treatments in the trisomic phosphoproteome

**Figure 3:**
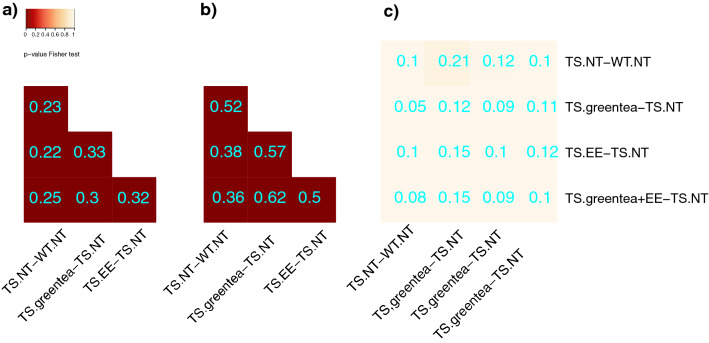
Overlap of the differentially abundant and differentially phosphorylated proteins in trisomic mice after different treatments. (**a**) Heatmap showing the overlap between differentially abundant proteins across the different treatments in transgenic mice. (**b**) Overlap between differentially abundant phosphopeptides across the different treatments. (**c**) Overlap between differentially abundant proteins and proteins with differentially abundant phosphopeptides. The color-code goes from very low p-values (red) to high p-values (yellow) of the exact Fisher test. TS, ts65Dn mice. WT, wild type mice. NT, not treated. Greentea, “EGCG-containing green tea extract”. EE, Environmental Enrichment. The Szymkiewicz-Simpson overlap coefficient is printed in cyan.

Our results show that the treatments (EGCG containing green tea extracts, EE and their combination) modulate common targets, many of which are altered in trisomic mice (Fig. 3). In fact, changes in the proteome and the phosphoproteome exhibit a significant overlap in all the different conditions tested (around 20–30% for the proteome and 50% for the phospho-proteome; p-value Fisher’s test < 0.05) (Fig. 3A,B).

Given that the changes observed in protein abundances showed minimal overlap with proteins changing their phosphorylation, the trisomy and pro-cognitive treatments affected different proteins in the proteome (abundances) and the phospho-proteome (phosphorylation levels) (Fig. 3C).

We used this *Ts65Dn’s network* to identify potential mechanisms of the pro-cognitive treatments by analyzing their capability to restore the deregulated nodes, both at the proteome and phosphoproteome level. We considered as “rescued”, those proteins altered in Ts65Dn mice compared to WT with abundances and/or phosphorylation levels restored partially or completely, i.e. 50–150% of the WT untreated values by at least one of the treatments (Tables 1, 2, Fig. 3 and Supplementary Fig. S5, for more details see “Methods”^7^).

**Figure 4:**
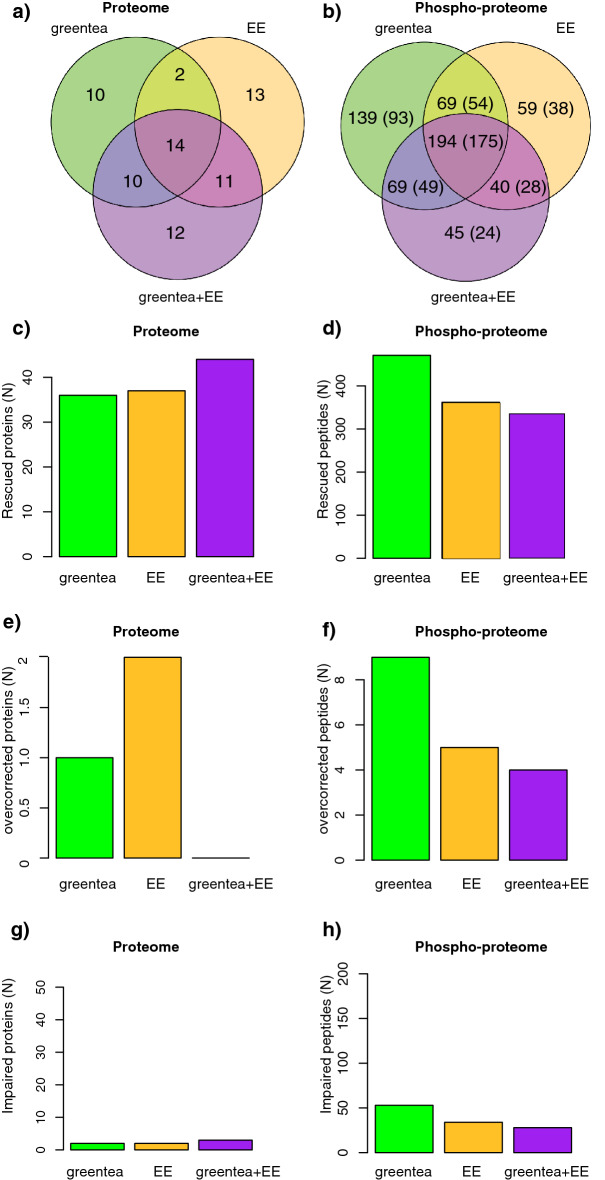
(Phospho-)proteomic alterations in trisomic mice are partially restored by the treatments. Venn Diagrams showing (**a**) the overlap of proteins that exhibit restored (rescued) abundances after the treatments, and (**b**) the overlap of phosphopeptides that exhibit restored (rescued) abundances after the treatments (in brackets the number of phosphoproteins). (**c–h**) Barplot showing the number of rescued proteins (**c**) or phosphopeptides (**d**), overcorrected proteins (**e**) or phosphopeptides (**f**), and impaired proteins (**g**) or phosphopeptides (**h**), after each of the tested treatments. TS, ts65Dn mice. WT, wild type mice. NT, not treated. greentea, “EGCG-containing green tea extract”. EE, Environmental Enrichment.

At the proteome level, we detected 57 proteins rescued by green tea and/or EE, of which 16 proteins were common to both treatments (Fig. 4A). More than half of these 57 proteins were also rescued by the combined treatment, whereas 12 proteins were found to respond only to the green tea + EE treatment. Similarly, when analyzing the rescued phosphopeptides (Fig. 4B), 570 phosphopeptides (corresponding to 437 proteins) were rescued by either green tea and/or EE, of which 208 (142 proteins) were specific to green tea, and 99 (66 proteins) to EE. The combined treatment rescued 53% of these phosphopeptides, and further rescued another 45 phosphopeptides (belonging to 24 proteins). The list of the “rescued proteins” can be found in Supplementary Table 21.

Altogether, these results show that green tea and EE had a number of additive and possibly synergistic effects, as their combination (green tea + EE) was different from the individual treatments. Green tea was the treatment rescuing more phosphosites, while green tea + EE was more effective at rescuing protein abundances (Fig. 4C,D). Interestingly only a small fraction of proteins was “overcorrected” or showed an even bigger difference after treatment when compared to “physiological” WT levels (Fig. 4E–H).

We then performed a gene ontology enrichment analysis of the proteins altered by the trisomy and rescued by the treatments, to better understand the molecular processes involved. When analyzing the rescued phosphopeptides, we detected significant enrichments in all the categories found deregulated at the level of the phospho-proteome (Supplementary Fig. S6, Supplementary Table 22).

We then analyzed how the rescued proteins were distributed along the *Ts65Dn network* (Fig. 2). Around 75% of the *seed* proteins were rescued by at least one of the treatments, while only 11% was overcorrected (more than 1.5-fold compared to WT untreated levels), not sufficiently rescued (less than 0.5 fold change), or further impaired. Interestingly, five out of the seven *seed* proteins that are hubs of the network–RPS27A , SOS1, CTNNB1, DLG4, and PLCG1–were rescued by at least one of the treatments, suggesting that the treatments are acting on key points in the *Ts65Dn’s network* (Table 5).

### Green tea, EE, and green tea + EE rescue the trisomic kinome

Interestingly, 3 of the 6 kinases that changed their abundance (1 downregulated, and 2 upregulated), were rescued by at least one of the treatments, including the rescue of the upregulation of Erk1. Most of the de-phosphorylated kinases (34/39) recovered their phosphorylation levels by at least one of the treatments. Overall, out of the 45 deregulated kinases, 11 were rescued by each of the three treatments, and 20 by at least two of the treatments. Green tea treatment was particularly efficient in rescuing kinases from the CAMK and TKL group, while most of the kinases of the AGC and CMGC group were rescued upon EE.

### Green tea, EE, and green tea + EE effects beyond the DS network

The proteins modified by the treatments included not only those altered in trisomic mice (40% of the overall proteins responding to treatments), but also 830 proteins that were not altered when comparing trisomic versus wild type mice. This indicated that the treatments were not simply restoring the portion of the proteome altered due to the trisomy, but had a much wider effect, affecting a total of 1397 proteins. In order to delineate the overall effect of the treatments we re-analyzed all the proteins responding to the treatments, and not only the rescued portion of the proteome. The gene ontology enrichment analysis detected categories related with “synapse”/neurodevelopment, chromatin/immune systems, microtubule/cytoskeleton, and GTPase activity similar to what we found for the subset of the rescued proteins (see above). Interestingly, these enrichments were lost when removing the rescued proteins. In fact, the non-rescued proteins were not enriched in any specific function when examined alone (Supplementary Fig. S6, Supplementary Table 23).

### The effects of treatment with green tea, EE, and their combination are genotype specific

We assessed if there was a genotype-specific (phospho-)proteome response to the different treatments, ie. Green tea, EE, and their combination. As detailed above, in trisomic mice the treatments were rescuing most of the nodes (Fig. 2). In WT over 74% of the nodes were modified by one of the treatments (Supplementary Fig. S7, dark green nodes) shifting the abundance and/or phosphorylation of these proteins in the *Ts65Dn’s network* towards levels similar to the ones observed in trisomic mice. As in trisomic mice, also in WT the treatments were affecting far more proteins than those altered by the trisomy, reaching a total of 1192 proteins changing their abundance and/or phosphorylation. GO-term analysis of these proteins showed once again similar enrichments than the ones seen in the trisomic hippocampus (Supplementary Fig. S6, Supplementary Table 23). Noteworthy, the fraction of these proteins with abundance levels shifted towards trisomic levels (dark green nodes in Supplementary Fig. 5) was enriched in synaptic pathways (Supplementary Fig. S6, Supplementary Table 23).

**Figure 5:**
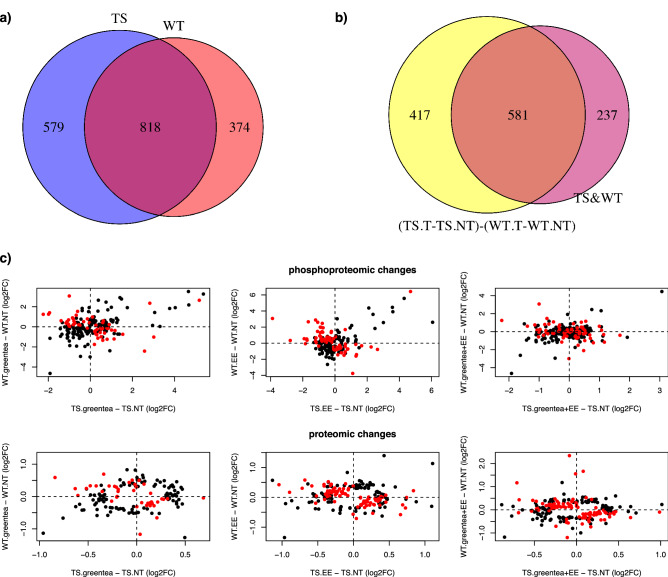
The effects of green tea extract, EE, and the combined treatment are genotype specific. Venn Diagram showing (**a**) the overlap between proteins changing their abundance or their phosphopeptide levels upon any of the treatments in TS and wild type mice; and (**b**) the overlap between the (phospho-)proteins having the same behavior upon one of the treatments in TS mice and WT mice, and (phospho-)proteins exhibiting a genotype-specific response to treatments. (**c**) Plot comparing phosphopeptide (top panel) and protein (bottom panel) fold changes upon each of the three treatments in TS mice (x-axis), and wild type mice (y-axis). Protein or phosphopeptides with a significant interaction in the contrast *(TS.T* − *TS.NT)* − *(WT.T* − *WT.NT)*, where T stands for one of the treatments, and NT for “not treated”, are indicated as red dots. TS, ts65Dn mice. WT, wild type mice. greentea, “EGCG-containing green tea extract”. EE, Environmental Enrichment.

Of the 1192 proteins responding to treatments in WT mice, 818 were common to the ones responding in trisomic mice (Fig. 5A, > 68% overlap, p < 0.001, Fisher Exact Test), and were enriched in the same GO-term categories (Supplementary S6, Supplementary Table 23). To understand if the treatment had genotype-specific effects, we investigated the proteins and phosphorylation sites changing in abundance upon the different treatments in WT compared to Ts65Dn mice (TS). Specifically, we compared the abundance changes observed in Ts65Dn mice upon treatment (Ts65Dn treated-Ts65Dn untreated, or TS.T–TS.U) with the abundance changes observed in WT mice upon treatment (WT treated-WT untreated, or WT.T–WT.U). Overall, we found that 998 proteins were responding significantly different to the treatments in Ts65Dn compared to WT mice. These (phospho-)proteins were enriched in categories including “synapse-related” and other neuronal components, as well as in categories associated with learning and memory (Supplementary Fig. S6, Supplementary Table 23). Of these 998 proteins, 581 were responding to the treatments both in WT and Ts65Dn mice (58% of the 998; Fig. 5B). Interestingly, when comparing the fold changes for each treatment in WT versus trisomic mice, we observed that most proteins and phosphopeptides differentially regulated by the treatments in trisomic mice, showed opposite changes in WT mice (red dots in the top-left and bottom-right portion of the plots, Fig. 5C). This observation suggests that the treatments have an opposite effect on their levels in the two genotypes.

### Part of the *Ts65Dn’s network* overlaps DYRK1A’s

In a previously reported proteomic analysis in mice overexpressing Dyrk1A we found that the pro-cognitive treatments were rescuing the *Dyrk1A* transgenic hippocampal protein network of approximately 70%^7^. We therefore compared the list of proteins rescued by green tea, EE, or both treatments in trisomic mice with those rescued in *Dyrk1A* transgenic mice. We found significant overlaps (all p-values < 0.01, Fisher exact test) with 52 proteins whose phosphorylation state was rescued by green tea, EE or their combination in both Ts65Dn and *Dyrk1a* overexpressing mice, though in different phospho-sites (Supplementary Fig. S8). The observation agrees with the fact that 26 seed proteins of the Ts65Dn disease network are known DYRK1A interactors (including the hub APP, SYNJ1, MAP1B, BRAF, GRIN2A, AMPH, STXBP1, and SF3B1) and 17 of them (including MAP1B, STXBP1 and BRAF) were rescued by at least of one the pro-cognitive treatments.

## Discussion

Environmental enrichment (EE) and the administration of green tea extracts containing epigallocatechin-3-gallate (EGCG) improve cognition in mouse models and individuals with Down syndrome, but the underlying mechanisms are unknown. We here shed light onto the possible mechanisms underlying the effects of green tea extracts, EE and their combination on the hippocampal proteome and phosphoproteome in a Down syndrome mouse model (Ts65Dn). We detected an alteration of both protein abundances and phosphorylation levels in the hippocampus of Ts65Dn mice that was not limited to triplicated genes but extended genome-wide affecting chromatin and neuronal-related categories. Interestingly, the treatment of Ts65Dn mice with green tea, EE, and their combination was able to rescue the *Ts65Dn’s network*.

### The (phospho-)proteome deregulation is not limited to chromosome 21

The Ts65Dn mouse model is considered one of the best models for DS because of its face validity (it recapitulates most of the cognitive and neural phenotypes detected in DS) and its wide use in previous reference studies^14^. It bears a translocation between chromosome 17 and 16 resulting in trisomy 16 and the triplication of approximately 2/3 of the genes orthologous to HSA21. As such, we would expect a prevalence of upregulated protein coded by the triplicated regions. However, while 48 proteins increased their abundance, even more proteins, 67, decreased their level. We detected 19 proteins whose orthologous map on HSA21 (HSA21 proteins). Of those, five were upregulated: APP, SOD1, CBR1, CBR3, and PCP4. This corresponds to 30% of the detected HSA21 proteins, a percentage much higher than other chromosomes that only reached maximum 10% of differentially abundant proteins (Fig. 1C–E). A concern related to the Ts65Dn model is that it bears some triplicated genes not orthologous to HSA21. However, out of these genes only the HSA6 protein ezrin was detected in our dataset, and this protein was not differentially abundant or phosphorylated (Table 2).

### Kinome and epigenomic deregulation in Ts65Dn mice

Regarding the phosphoproteome, we detected more changes in phosphopeptide levels than in protein abundances. . Interestingly, when analyzing the proteins to which these phosphopeptide belonged, we found many in common with previous findings^15^, such as DLGAP4, ARVCF, GRIN2A, PCLO, DLG1, DYNC1LI1, SPI1L1, RTN4, ADD2, and SYNJ, involved in synaptic processes (transport, signalling, assembly), microtubule, and glutamate receptors.

These changes in phosphorylation levels are possibly associated with the detected differences in abundance and phosphorylation state in 44 de-regulated kinases (6 with alteration in abundance, and 38 with altered phosphorylation). These proteins belonged to different kinase families but we detected the consensus motif *PXSP* as the most common phosphorylation site. This is a sequence recognized by mitogen-activated protein (MAP) kinases, which is important for learning and memory^16^.

EE, green tea extracts and their combination were able to rescue half of the kinases with altered abundances, and over 87% of the ones with altered phosphorylation, with green tea rescuing mainly CAMK and TKL kinases, and EE, AGC and CMGC kinases.

To better understand the concerted effects of proteins changing their abundance and the ones changing their phosphorylation levels in trisomic mice we analyzed them together in a PPI *Ts65Dn’s network*. The proteins in the network were functionally related, as suggested by their number of interactions, higher than expected by chance. When analyzing the main hubs of the Ts65Dn network we found many proteins related to Down syndrome. The protein with most interactions was ubiquitin (RPS27A), and the disturbance of the polyubiquitination machinery is a key feature of Down syndrome neurodegeneration with accumulation of polyubiquitinated toxic proteins^17^. Another interesting protein is SOS1, that was less phosphorylated in Ts65Dn mice, and was rescued by EE treatment. It promotes Ras activation in the MAPK/ERK pathway and is involved in axon guidance. Interestingly, mutation in this gene leads to a developmental syndrome called Noonan syndrome^18^ which shares hallmarks with Down syndrome like mildly unusual facial characteristics, short stature, and heart defects. Also, CREB binding protein (CBP) phosphorylation was found only in trisomic samples and it was rescued by the treatments. This protein is an histone acetyl transferase that facilitates the transcription of CREB dependent genes, such as the immediate early genes activated during cognitive processes. Mutations in the CREBBP gene cause Rubinstein-Taybi syndrome, a condition^19^ that, similar to Down syndrome, is characterized by short stature, moderate to severe intellectual disability, and distinctive facial features. As expected, APP abundance was increased in trisomic samples, and it was a hub in the Ts65Dn network. *APP* is one of the most important Down syndrome candidate gene, whose triplication is responsible for neurodegeneration and early onset Alzheimer disease in individuals with Down syndrome. Another hub of the network, Catenin Beta 1, was found phosphorylated only in trisomic mice and rescued upon our treatments. Interestingly the Wnt/β-catenin pathway is downregulated in the hippocampus of adult DS individuals with Alzheimer’s disease^20^. DLG4, a scaffolding protein present in the post-synaptic density implicated in glutamatergic synaptic functions^21^, was found differentially phosphorylated in Ts65Dn and rescued by our treatments. Finally, Phospholipase C, gamma 1 phosphorylation was not detected in trisomic samples but was present after treatment. APP, CREBBP, DLG4 formed independent modules in the network, while SOS1, beta-catenin, ubiquitin and PLCG1 formed a fourth module, indicating that they were implicated in common molecular mechanisms.

### Treatment effects on the hippocampal proteome and phosphoproteome

Since green tea-extracts and EE improve cognition in Ts65Dn mice^1,2^ we wanted to elucidate the molecular mechanisms responsible for these improvements.

In our (phospho-)proteomic profiles, green tea extracts and EE not only rescued five out of the seven seed protein hubs but also more than 70% of the seed proteins. We detected five main categories that were compromised in trisomic mice and rescued by our pro-cognitive treatments: chromatin-related, neuronal projection/neuron development, microtuble/cytoskeleton, synapsis/cognition, and GTPase/Kinase activity.

Interestingly, green tea extracts rescued mainly protein phosphorylation, while the combination of green tea extracts and EE acted more on protein abundance. Previous studies had shown that both green tea extract with EGCG and environmental enrichment inhibit the tyrosine kinase DYRK1A^5^, which regulates the phosphorylation of many important proteins in the brain^2,22^. In a previous study^7^, we showed that both treatments could similarly rescue 70% of the Dyrk1A transgenic hippocampal (phospho-)protein network. Interestingly, 26 DYRK1A targets were seed proteins of the Ts65Dn network and more than 65% of these proteins were rescued by one of the pro-cognitive treatments. In both Dyrk1A transgenic mice and Ts65Dn mice the phosphorylation levels of 52 proteins were rescued by green tea, EE or their combination (Fig. S8).

An interesting mechanism revealed by our phosphoproteomic study was a re-balancing of the epigenetic state, which is compromised in Down syndrome^23^. Strikingly we observed a reduction in the levels of global histone H3 acetylation in the hippocampus of Ts65Dn mice, suggesting a more repressed chromatin that might contribute to a reduced expression of memory promoting genes , as previously proposed^24^.

Regarding our treatments, EE is known to trigger activity dependent transcription with activation of the transcription factor CREB and increase of global acetylation that favours cognition^25^. Moreover, EGCG shows a histone deacetylase inhibitor^26,27^ and DNA methyl transferase activity^28^. This hypothesis is corroborated by fact that among the impaired and rescued categories we found many chromatin-related molecular categories.

Another important question was the possible interaction between treatments, as previous behavioral studies have shown that the effect of the combination of green tea and environmental enrichment is more potent than each treatment alone^12,13^. Our (phospho-)protemic experiments revealed that the effects of the combination were different than the ones of the single treatments, indicating a strong interaction. Moreover, their effects extended far beyond the (phospho-) proteins impaired by the trisomy, although these “out of the network” proteins were enriched in similar categories. This indicates that green tea, EE and their combination not only rescue the deregulated proteins but also related proteins beyond the Ts65Dn network with a possible positive compensatory mechanism. We speculate that this would contribute to the improvements in cortico hippocampal-dependent learning and memory in trisomic mice once treated with green tea and environmental enrichment^13^. Unfortunately, the present experiments do not allow to directly correlate our proteomic and phospho-proteomic findings with cognitive levels in the same mice. However our previous study showed that, the combined treatment with EE and Green tea extracts improved corticohippocampal-dependent learning in both young and middle age Ts65Dn mice^12,13^. Conversely in DYRK1A transgenic mice the combined treatment did not show better results compared to EGCG alone^7^.

Our pro-cognitive treatment had a different effect on wild type mice than in Ts65Dn mice, confirming our previous observations in DYRK1A transgenic mice^7^. Around 44% of the proteins changing upon pro-cognitive treatments were common in wild type and trisomic mice, but with an opposite direction of change (Fig. 5). As such, over 70% of the nodes modified by the treatment(s) in WT hippocampi shifted their abundance and phosphorylation towards levels similar to the ones observed in trisomic mice. These changes could be interpreted as deleterious, and indicate that further studies are needed to optimize the treatment efficacy of green tea and EE.

## Conclusion

The pro-cognitive potential of green tea extracts and environmental enrichment was already shown in previous works^1,2,5,7,12,13^, but the molecular effect on the proteome and phosphoproteome remained elusive. We here show that these pro-cognitive treatments are able to restore more than 70% of the protein deregulation in trisomic mice, and induce compensatory mechanisms by acting on proteins of the same categories. Our downstream analyses indicate as possible mechanisms both the re-establishment of a proper epigenetic state and the rescue of the kinome deregulation, possibly initiated by triplication of key genes such as DYRK1A. In light of these results, promising combinatorial therapies will boost or prolong current cognitive-enhancement for the treatment of intellectual disabilities.

## Methods

### Animal models

We used female mice from wild-type (WT) and Ts(1716)65Dn (Ts65Dn) mice, obtained through crossings of a B6EiC3Sn a/A-Ts (1716)65Dn (Ts65Dn) female to B6C3F1/J males (from The Jackson Laboratory, Bar Harbor, USA). Mice were bred in the Animal Facilitity of the Barcelona Biomedical Research Park (PRBB, Barcelona, Spain, in standard cages (20 × 12 × 12 cm Plexiglas). Animals were kept in small groups (2–3 animals) in a 12-h light–dark cycle (8:00–22:00 h), with constant humidity (60%) and temperature (22 ± 1 °C) with ad libitum access to food and water. Procedures were approved by the local ethical committee (Comité Ético de Experimentación Animal del PRBB (CEEA-PRBB); MDS-16-0035PR1-P2), meeting the guidelines of the local (law 32/2007) and European regulations (EU directive e no. 86/609, EU decree 2001-486) and the Standards for Use of Laboratory Animals no. A5388-01 (NIH). The CRG received autorization to work with genetically modified organisms (A/ES/05/I-13 and A/ES/05/14).

### Experimental design

We used 2 months trisomic (TS) and wild type (WT) mice. Animal were whether left non-treated (NT) or treated with three different protocols:

- Administered with green tea extract (greentea).
- Reared under enriched environment (EE) conditions.
- Both administered with green tea extract and reared under EE (greentea + EE).

We used five mice per group, randomly selected from each cage (40 total mice).

### Pro-cognitive treatments

In order to prepare green tea extract (Mega Green Tea Extract, Decaffeinated, Life Extension, USA; EGCG content of 326.25 mg per capsule), we prepared the solution every 2–3 days in water at 0.33 mg/ml corresponding. This correspond to an average dose of 42 mg/Kg per day for one month.

For EE we used Plexiglas cage (55 × 80 × 50 cm) with toys, and platforms of different shapes, sizes, colors and textures, changing their arrangement every 3 days to maintain the novelty conditions. To increase social interactions we house 6–8 mice per cage, instead of 2–3, rearing them just after the weaning period, for one month, and before sexual maturity, to reduce territorial aggressiveness^29^, the animals were reared in the same cage just after the weaning period, before they reached sexual maturity.

### Proteomics sample preparation

Dissected hippocampi were flash-frozen at -80 °C and then processed at the same time. We used RIPA-modified buffer for the homogeneization (50 mM tris–HCl pH 7.5, 150 mM NaCl, 1 mM EDTA, 1% NP-40, 0.1% sodium deoxycholate 5 mM b-glycerophosphate, 10 mM sodium fluoride, 10 mM sodium orthovanadate, and protease inhibitors from the Complete Protease Inhibitor Cocktail Roche).

Samples were sonicated with Bioruptor (Diagenode) for 5 min with 30 on/off cycles, on ice, and centrifuged for 10 min 10,000 rpm at 4 °C. The supernatants, containing proteins, were precipitated overnight at − 20 °C by adding six volumes of ice-cold acetone. We solubilized the acetone-precipitated proteins in a denaturation buffer (6 M urea and 200 mM ammonium bicarbonate in water. BCA-quantified proteins (Pierce), were reduced with dithiothreitol (DTT, 10 mM, 37 °C, 60 min), and alkylated with iodoacetamide (IAM, 20 mM, 25 °C, 30 min). We diluted samples with 200 mM ammonium bicarbonate up to 2 M urea, digested overnight with Lys-C at 37 °C, and then diluted two times and digested overnight with trypsin at 37 °C. Peptides were desalted using a C18 MicroSpin 300A silica column (The Nest Group Inc), dried using a speedvac, and dissolved in 0.1% formic acid in water.

### Titanium dioxide (TiO_2_) phosphopeptide enrichment

Titansphere chromatography was used to enrich phosphopeptides as described previously^7^. Tryptic peptides were desalted, completely dried, and dissolved with 100 μl of Loading Buffer [80% ACN (vol/vol) and 6% TFA (vol/vol)] at ~ 1 μg/μl. Samples went through a constricted TiO_2_ loaded spin tip, previously equilibrated with Loading Buffer. To achieve a complete binding we applied 2 × 50 μl and used a centrifuge at ~ 50 g. We washed the TiO2 spin tip with 50 μl of Loading Buffer and with 50 μl of Washing Buffer [50% ACN (vol/vol) and 0.1% TFA (vol/vol)]. Finally, we eluted teh phosphopeptides from the TiO2 spin tip with 30 μl of Elution Buffer (85% NH3-H2O, pH 11.0) into a tube containing one volume of 20% formic acid. We performed a second elution in the same tube with 3 μl Elution Buffer 2 [(80% ACN (vol/vol) and 2% formic acid (vol/vol)]. The eluted phosphopeptides were dried and dissolved with 0.1% formic acid in water for being analyzed by mass spectrometry (MS).

### Liquid chromatography-tandem mass spectrometry

2 μg of tryptic peptides from digested hippocampal tissue and phospho-enriched peptides from 100 μg of the same tissue were injected in an LTQ-Orbitrap Velos Pro mass spectrometer (Thermo Fisher Scientific) coupled to a nano-LC (EASY-nLC, Proxeon), for each samples. The orbitrap system’s performance was previously assessed showing coefficients of variations below 20% in single-shot technical replicates (from HeLa cells) for both proteome and phosphoproteome quantification^30^. Nano-LC was equipped with a reversed-phase chromatography column (of 25 cm) with an inner diameter of 75 μm, with 3 μm C18 particles (Nikkyo Technos, NTCC-360/75–3-25L), and a Nano Trap Column Acclaim PepMap100 100 μm × 2 cm C18, 5 μm, 100A (Thermo, 164199). The chromatographic gradients started at 93% of buffer A and 7% of buffer B with a flow rate of 250 nL/min during 5 min and linearly changed to 65% buffer A and 35% buffer B after 4 h. The column was washed for 16 min with 90% buffer A and 10% buffer B (Buffer A: 0.1% formic acid in water. Buffer B: 0.1% formic acid in acetonitrile) after each analysis.

The mass spectrometer was used in positive ionization mode with the nanospray voltage set at 2.2 kV and source temperature of 250 °C. We used the Ultramark 1621 for the FT mass analyzer for external calibration prior to the analyses. We used the background polysiloxane ion signal at m/z 445.1200 as lock mass. We operated the instrument in data-dependent acquisition (DDA) mode with 1 microscan at resolution of 60,000 at 400 m/z. Survey scans were recorded over a mass range of m/z 350−2000 with detection in the Orbitrap mass analyzer. We sat the auto gain control (AGC) to 106, dynamic exclusion to 60 s, and we activated the charge-state filter disqualifying singly charged peptides for fragmentation. Following each survey scan, We selected for fragmentation the top 20 most intense ions with multiple charged ions above a threshold ion count of 5,000 at normalized collision energy of 35%. We acquired in the linear ion trap Fragment ion spectra produced via collision-induced dissociation (CID) and collision-induced dissociation MultiStage activation (CID MSA) for proteome and phosphoproteome, respectively. AGC was set to 5 × 104 and isolation window of 2.0 m/z, activation time of 0.1 ms and maximum injection time of 100 ms.

### Mass spectrometry data analysis

We acquired and processed the mass spectra were processed with the MaxQuant computational platform version 1.5.2.8^31^ (RRID:SCR_014485). The MS2 spectra were searched with the Andromeda search engine^32^ against the Uniprot sequence database for Mus musculus (17,263 forward entries; version from July 2015). The search included cysteine carbamidomethylation as a fixed modification, and N-terminal protein acetylation and methionine oxidation as variable modifications. For the phosphoproteome analysis, we also added the phosphorylation on serine, threonine and tyrosine as variable modifications. We allowed a maximum of two mis-cleavages, 4.5 ppm as mass tolerance for precursor ions, and 0.5 Da as mass tolerance for fragment ions. The fraction of false discovery rate was set to 0.01 at both the peptide and protein level, and protein identification required at least one unique or razor peptide per each protein group.

We used MaxQuant algorithm to retrieve accurate extracted ion currents (XICs) per each peptide feature for quantification purposes. We calculated for each peptide the areas under the curve. For the statistical analysis we used the R package MSstats version 2.6.0^33^ (RRID:SCR_014353). In some of the experimental replicates we excluded from the anaysis one of the five biological replicates, when the number of peptides identified was substantially lower than the average of the whole experiment (e.g. < 1000 peptides). Specifically: one replicate was excluded from the TS.greentea + EE and WT.EE groups in the proteome analysis, and another replicate was excluded from the TS.greentea and WT.greentea + EE groups in the phosphoproteome analysis. To ensure high confidence in our quantitative data, we only used peptides observed at least in three of the five biological replicates (or at least in two when we remained only with four biological replicates), and no imputation of missing values was performed.

### Differentially expressed proteins and phosphopeptides

These analyses were peformed similarly to what we already described previously^7^. We performed the differential expression analysis with the MSstats package that is based on a family of linear mixed-effects models^33^. All downstream bioinformatics analyses were performed on proteins and phosphopeptides showing a significant change in abundance (Benjamini adjusted p-value lower than 0.05 and a log2(Fold Change) (log2FC) > 0.3 or < − 0.3). For the phosphoproteomic analysis, we considered only phosphorylation sites with a localization probability of 0.5 to avoid uncertain sites. We added to the lists of differentially abundant proteins and phosphopeptides the proteins or phosphopeptides uniquely present in one condition of the ones compared. A peptide was defined as “absent/low abundant” when it was detected in less than 0, 1 or 2 out of 5 biological replicates for a given condition (or less than 2 out of 4 biological replicates). Proteins belonging to the GO-term cell component “blood microparticle” were filtered out from all datasets before proceeding with downstream analyses, since blood contamination of the samples is a common problem.

We computed the following contrasts (in log2 fold changes) for each of the treatments (TS: TS mice; WT: wild type mice; NT: not treated mice; T, treated mice: whether TS.greentea, TS.EE, or TS.greentea + EE):

- TS.NT − WT.NT (deregulated proteins in untreated TS mice).
- TS.T − TS.NT (proteins responding to the treatments in TS mice).
- WT.T − WT.NT (proteins responding to the treatments in WT mice).
- (TS.T − TS.NT) − (WT.T − WT.NT) (interaction: proteins responding differently to the treatments in TS compared to WT mice).

For each protein/phosphopeptide whose abundance was significantly changing in the TS.NT − WT.NT we first calculated the log2-fold-change obtained in the contrast TS.NT − WT.NT (genotype gap). Thereafter, we computed the fold-change observed in the TS.T − WT.NT (treatment gap). We therefore calculated the fraction of recovered abundance, both in protein or phosphopeptides levels, after treatment—given by the difference between the genotype gap and the treatment gap—on the genotype gap: (genotype gap) – (treatment gap)/(genotype gap) (percentage of recovery).

When WT.NT is set to 0 (log2(1) = 0):

- TS.NT − WT.NT can be written as TS.NT – log2(1) = TS.NT-0 = TS.NT.
- TS.T − WT.NT is equal to the levels impaired at the basal state + levels after treatment = (TS.NT − WT.NT) + (TS.T − TS.NT) = TS.NT-0 + TS.T –TS.NT = TS.T.

And therefore:

(TS.NT − TS.T)/TS.NT = [(TS.NT − WT.NT) − (TS.NT − WT.NT) + (TS.T + TS.NT)]/(TS.NT − WT.NT).

The fraction of recovery could span from 0 (no recovery) to 1 (100% recovery). A value > 1 suggests overcorrection, while a value < 0 an impairment.

We defined as “rescued protein” the proteins, significantly changing (at the abundance or phosphorylation levels) inTS.NT-WT.NT, with a percentage of recovery from 50 to 150%; as “overcorrected proteins”, the ones with a percentage of recovery > 150; “not sufficiently rescued” those proteins with a percentage of recovery between − 50% and 50%; and “impaired proteins” those with a percentage of recovery < − 50%.

Similarly , we calculated the “percentage of impairment” by using in place of the TS.NT − WT.NT contrasts, its reverse WT.NT − TS.NT, to compute how much the treatment in the wild type was reducing the differences between TS and WT.

### DYRK1A interactors

Given that previous work had identified EGCG as a potent DYRK1A inhibitor, we also explored DYRK1A interactors, taken from the mammalian verified interactors reported in previous literature^6,34–36^. We assessed the significance of the overlaps using a Fisher Exact Test.

### Network analysis

To explore the functional significance of the differentially expressed and phosphorylated proteins, we built a protein–protein interaction network, in which the differentially abundant proteins and the proteins changing their phosphorylation status in TS vs WT were expanded to their direct interactors.

For the expansion, we used a list of bona fide physical interactors coming from the STRING datbase (version 10)^37^ and an internal database from Interactome3d version 2017_01^38^. We only considered interactions with a very high score (> 0.9) in STRINGdb and IMEx index^39^.

The visualization of the network was performed with the igraph R package^40^, using the Davidson Harel layout algorithm^41^. We used the ppi_enrichment function from the STRINGdb package^42^, to compute the odds of the observed number of the interaction compared to the expected ones.

To compute differences in the properties of the network between different sub-graphs of the network (i.e. differences in node degree) we permuted 1000 times the network, recalculating at each step the given properiesy on a random subset of the same size.

### Enrichment analyses

We annotated our proteins with the R packages UniProt.ws and biomaRt^43^ (RRID:SCR_002987). We assessed the significance of the overlaps using a Fisher’s Exact test, considering as background the detected proteins specific for each contrast. We performed Gene Ontology Enrichment analysis with the clusterProfiler R package^44^ (RRID:SCR_016884).

### Identification of significant hubs

We analyzed the heavy tail distribution of interactions with the poweRlaw R package^45^, comparing the power-law, Poisson, exponential, and log-normal distribution. We then used the fitted distribution for calculating the p-values associated with each protein corresponding to find by chance a higher number of interactions per protein than the given protein, setting the threshold to define hub at a p-value < 0.05.

### Western blotting

Histones were isolated from whole hippocampi by acid-extraction from n animal, and for APP hippocampal proteins were extracted from 5 wild type and 5 Ts65Dn mice. After adding 6 × laemmli buffer to isolated histones or proteins stored in 2 M urea performed gel eletrophoresis and transferred the fractionated proteins to PVDF (only for histones) or nitrocellulose membranes using iBlot 2 (Thermofisher). The membranes were then blocked with 5% milk in TBS-Tween 0.1% (TBS-T) for 60 min before immunodetection with anti-diacetyl lysine 9 and lysine 14 histone H3 (AcH3K9K14) antibody (Cell Signaling Technology, #9677, RRID:AB_1147653) at a 1:1000 dilution, anti-histone H4 (Millipore 07-108, RRID:AB_2279758) at 1:500 dilution, anti-Actin at 1:2000 dilution (Sigma-Aldrich Cat# A2066, RRID:AB_476693), and anti-Amyloid Precursor Protein (Millipore Cat# AB5352, RRID:AB_91793) at 1:500 over-night at 4 ºC. Primary antibody incubation was followed by three washes (10 min, RT) in TBS-T before incubation with IRDye 800CW Goat anti-Rabbit IgG antibody (LI-COR Biosciences Cat# 925-32211, RRID:AB_2651127), three washes and visualization using Odissey Platform (LI-COR). Western blot’s bands were quantified by densitometry using ImageStudio.

Independent western blotting experiments were integrated by median normalization, excluding extreme values and statistical differences in the ratio AcH3/H4 or APP/ACTB were assessed with a linear mixed-effect model allowing for nested random effects (for sample coming from the same mouse).

## Supplementary information

Supplementary Figures.

Supplementary Legends.

Supplementary Table 1.

Supplementary Table 2.

Supplementary Table 3.

Supplementary Table 4.

Supplementary Table 5.

Supplementary Table 6.

Supplementary Table 7.

Supplementary Table 8.

Supplementary Table 9.

Supplementary Table 10.

Supplementary Table 11.

Supplementary Table 12.

Supplementary Table 13.

Supplementary Table 14.

Supplementary Table 15.

Supplementary Table 16.

Supplementary Table 17.

Supplementary Table 18.

Supplementary Table 19.

Supplementary Table 20.

Supplementary Table 21.

Supplementary Table 22.

Supplementary Table 23.

Supplementary Table 24.

Supplementary Table 25.

Supplementary Table 26.

## Data Availability

We deposited the mass spectrometry proteomics to the ProteomeXchange Consortium via the PRIDE^46^ partner repository (RRID:SCR_003411) with the dataset identifier EBI-PRIDE Accession PXD018515. We also created a repository with the R markdown file with the code to fully reproduce the analysis, the data showing that water intake was not altered when the treen tea extracts were dissolved in it, and the original images of the western blots: https://bitbucket.org/ilario_de_toma/proteomics_ts65dn.
