# Supplementary material for: Re-establishment of the epigenetic state and rescue of kinome deregulation in Ts65Dn mice upon treatment with green tea extract and environmental enrichment
Source: Sci Rep. 2020 Sep 29;10:16023. doi: 10.1038/s41598-020-72625-z (PMC7524756; doi:10.1038/s41598-020-72625-z)
Supplement: Supplementary file 1 — Supplementary Figures. [file 41598_2020_72625_MOESM1_ESM.pdf]

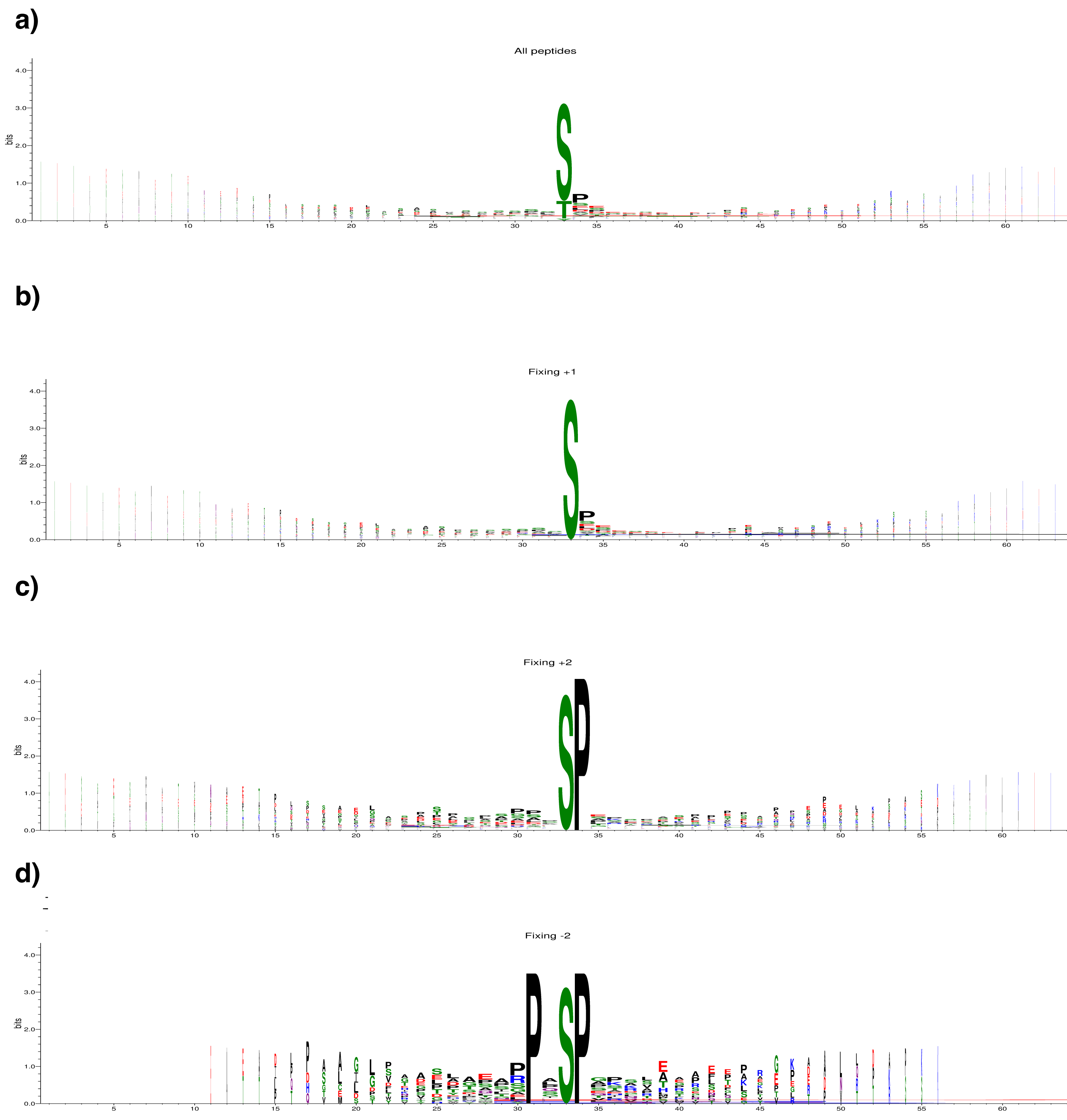

Supplementary Figure 1: Consensus motif of phosphopeptides altered in Ts65Dn. a) Sequence consensus motif in upregulated phosphopeptides (or exclusively present) in Ts65Dn mice compared to WT mice. The overall height of the stack indicates the sequence conservation at a given position, while the height of symbols within the stack indicates the relative frequency of each amino acid at that position. Polar amino acids are in green, neutral in purple, basic in blue, acidic in red, and hydrophobic in black. Stack width is scaled by the fraction of amino acids in the column. b) All phosphopeptides in a) with a phosphorylated Serine, the most common site of phosphorylation (502). c) All phosphopeptides in b) with a Proline in position +1, the most common +1 site (176). d) All phosphopeptides in b) with a Proline in position -2, the most common -2 site (43).

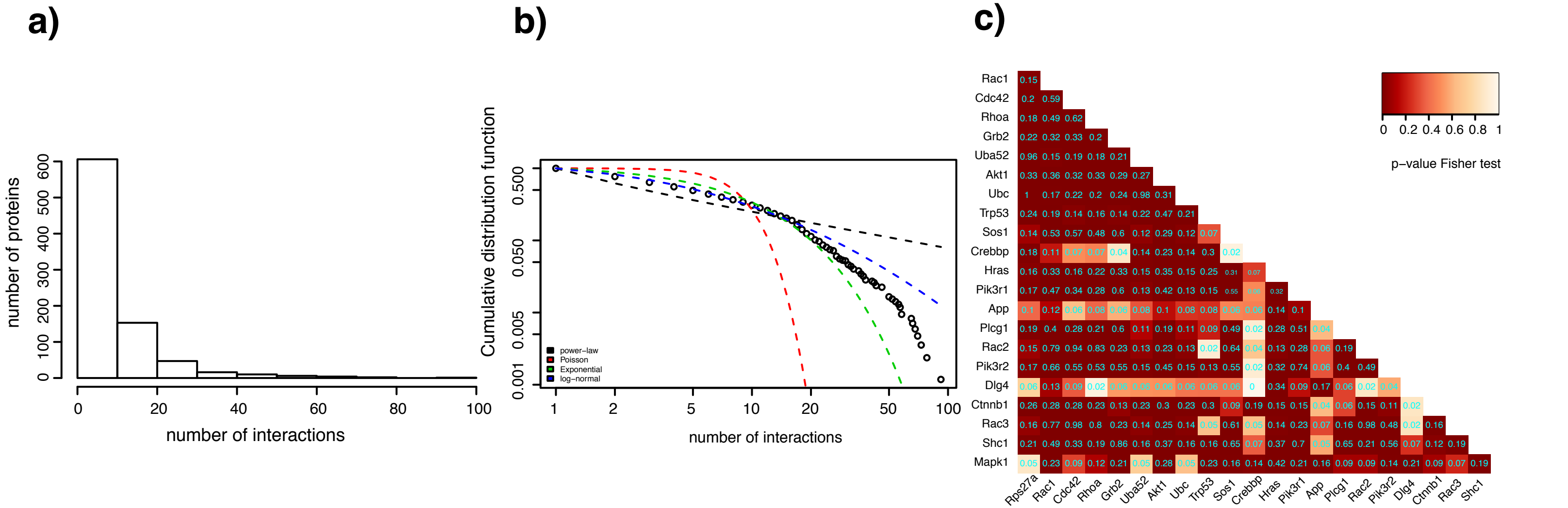

Supplementary Figure 2: Analysis of protein hubs in the Ts65Dn network. a) Histogram of the number of interactions per protein in the network. **b)** Cumulative distribution functions (y-axis) versus the number of observed interactions. Dashed lines indicate the theoretical distributions inferred by the observed data. **c)** Heatmap showing the results of the Fisher test comparing the overlap among the interactors of each of the hubs in the network. The percentage of overlap is printed out in cyan. Color code from red (significant) to yellow (not significant based on the p-value of the Fisher Test.

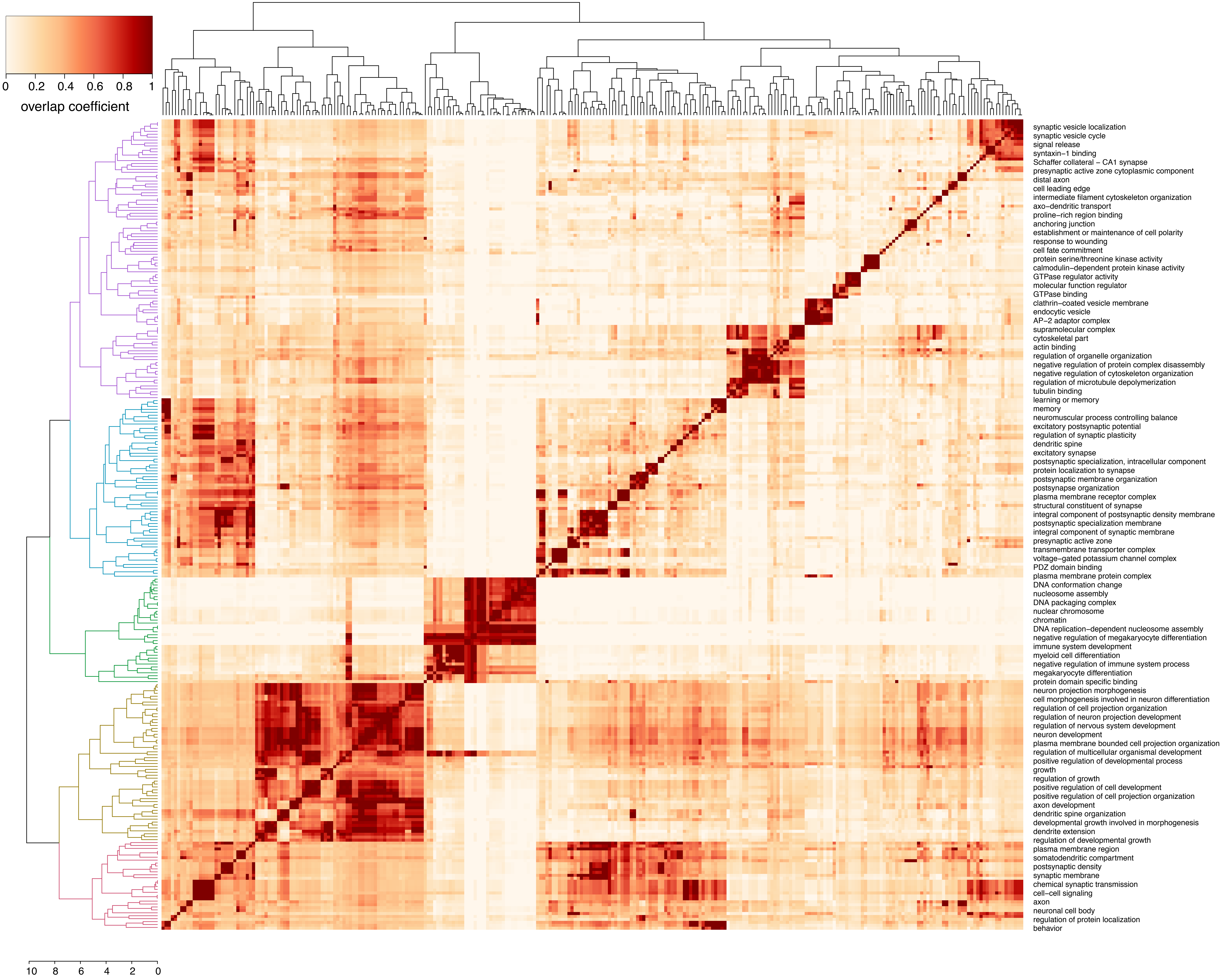

Supplementary Figure 3: Gene Ontology analysis of proteins significantly changing in Ts65Dn mice, and of proteins that are rescued (or not) after the treatment(s). Heatmap showing the Szymkiewicz–Simpson overlap coefficient among the significant categories. Hierarchical clustering reveals that the categories can be clustered in three main clusters, underlined with different colors.

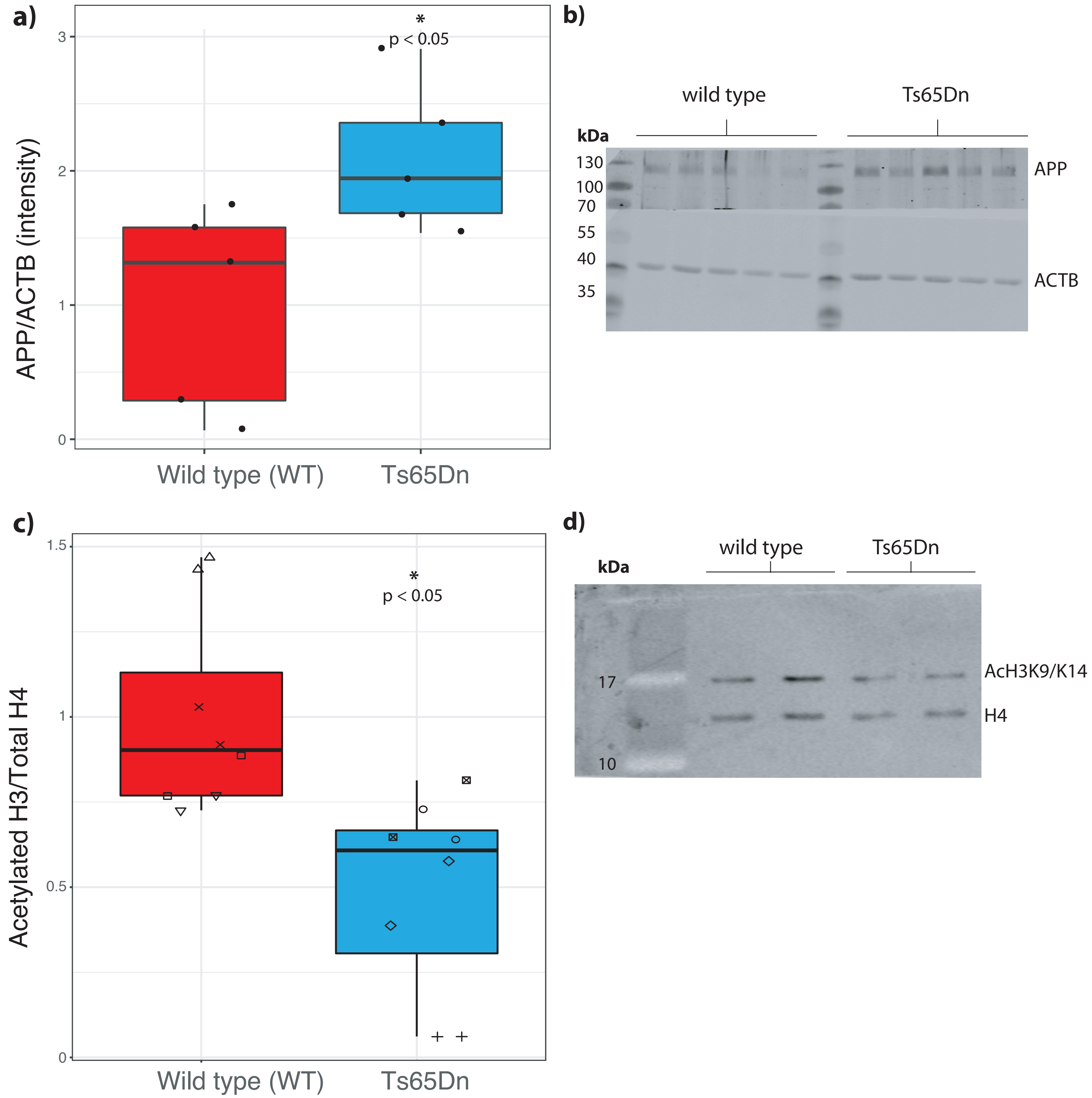

Supplementary figure 4. Ts65Dn mice show increased APP and reduced global histone acetylation level in the hippocampus. a) Boxplots showing the densitometric values for APP normalized to beta-actin ACTB). b) Western blot for APP and ACTB. APP levels are shown relative to WT (normalized as 1) c) Densitometric ratios normalized to total H4. Trisomic Ts65Dn mice show a ~25% reduction of global histone acetylation. Acetyl-H3 (Lys9 /Lys14) levels are shown relative to WT (normalized as 1). Technical replicates are represented with the same shape. TS indicates trisomic Ts65Dn. N=4 per genotype and age Western blotting analysis of Acetyl-H3 (Lys9 /Lys14) levels on histones from whole hippocampi. d) Representative Western blots for AcH3K9/K14 and histone H4.

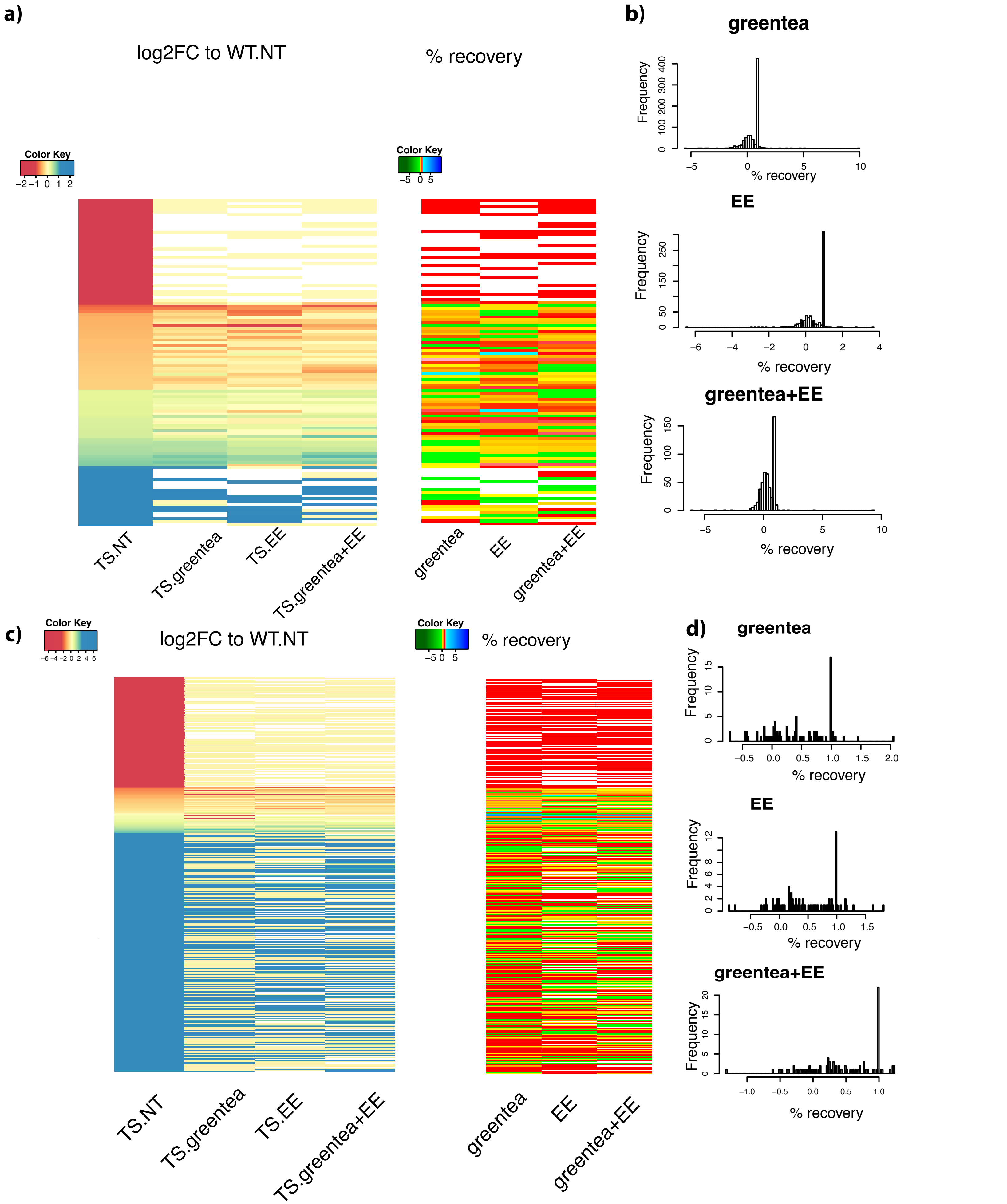

Supplementary Figure 5: Rescued proteins by the treatments in Ts65Dn mice. a) Heatmap of protein abundances that significantly change between trisomic and wild-type mice. Left panel. log2-fold-change values of protein abundance changes relative to wild type levels (orange= "downregulated", blue/green= "upregulated"). White: NA. Light yellow values indicate rescued level (log2FC to WT.NT is near 0). On the right: impairment in levels: proportional gradient of green. No significant effect: yellow towards orange. 50-]100% recovery: orange towards red. 100% -150% recovery: red towards pink. Overcorrection >150% cyan towards blue. Positively correlating proteins: red bars on the left side. Negatively correlating proteins: blue bars on the left side. **b)** Histogram showing the distribution of the percentage of recovery for each of the three treatments. **c)** As in (a) but for phosphopeptides. **d)** as in b) but for phosphopeptides.

a)

b)

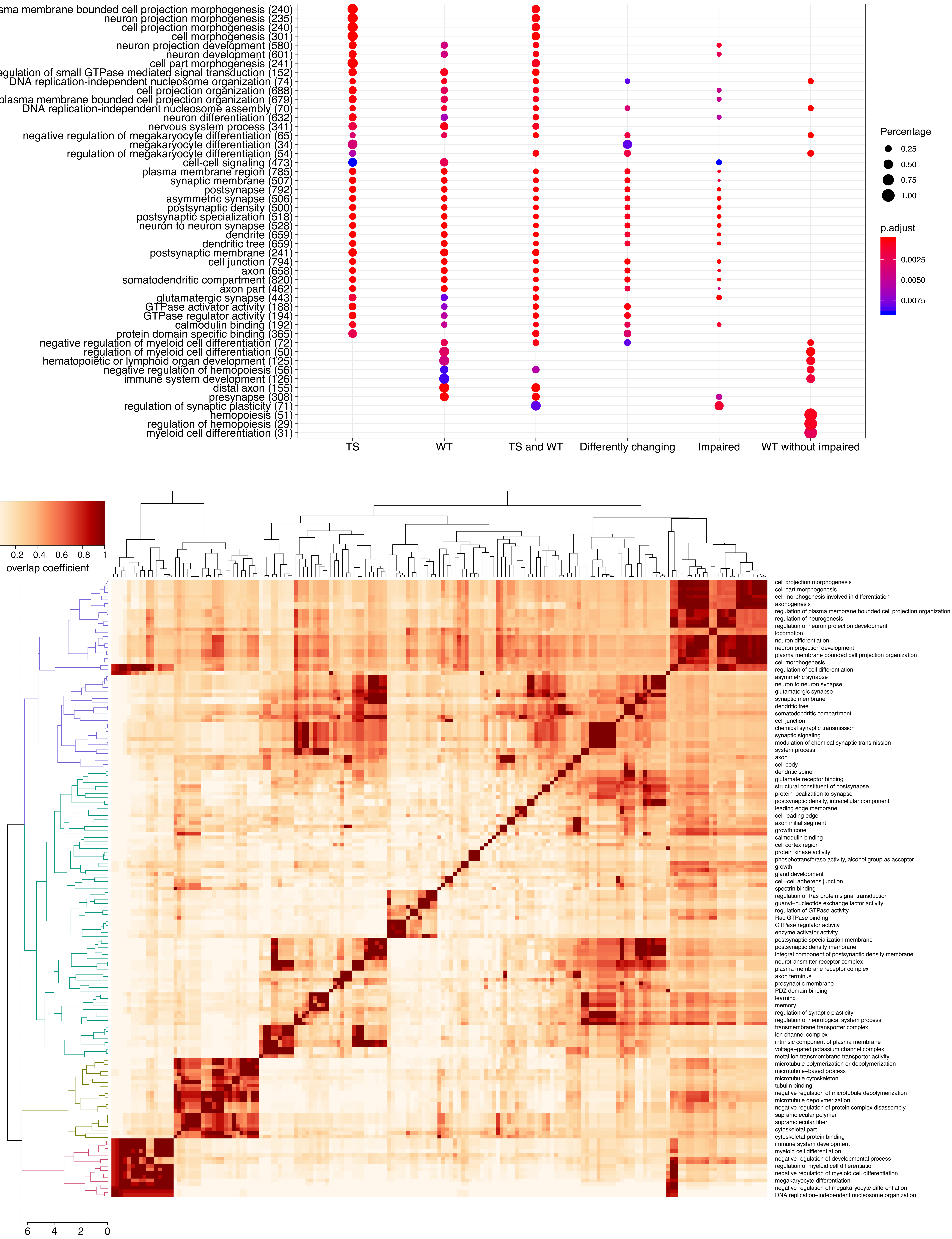

Supplemental Figure 6: Gene Ontology analysis of genes changing in wild type and/or Ts65Dn mice upon treatment. a) Enrichment analysis for biological processes, molecular functions, and cell components. The color-gradient indicates the adjusted p-value for the enrichment. Numbers in parentheses indicate the number of identified proteins in each category. Dot size corresponds to (protein count for each group)/(total protein count for each category). Only categories with an FDR < 1% are shown. **b)** Heatmap showing the Szymkiewicz–Simpson overlap coefficient among the significant categories in a). The number of EntrezID overlapping between a pair of categories is printed in cyan. Hierarchical clustering reveal that the categories can be clustered in four main "macro-categories", underlined with different colors.

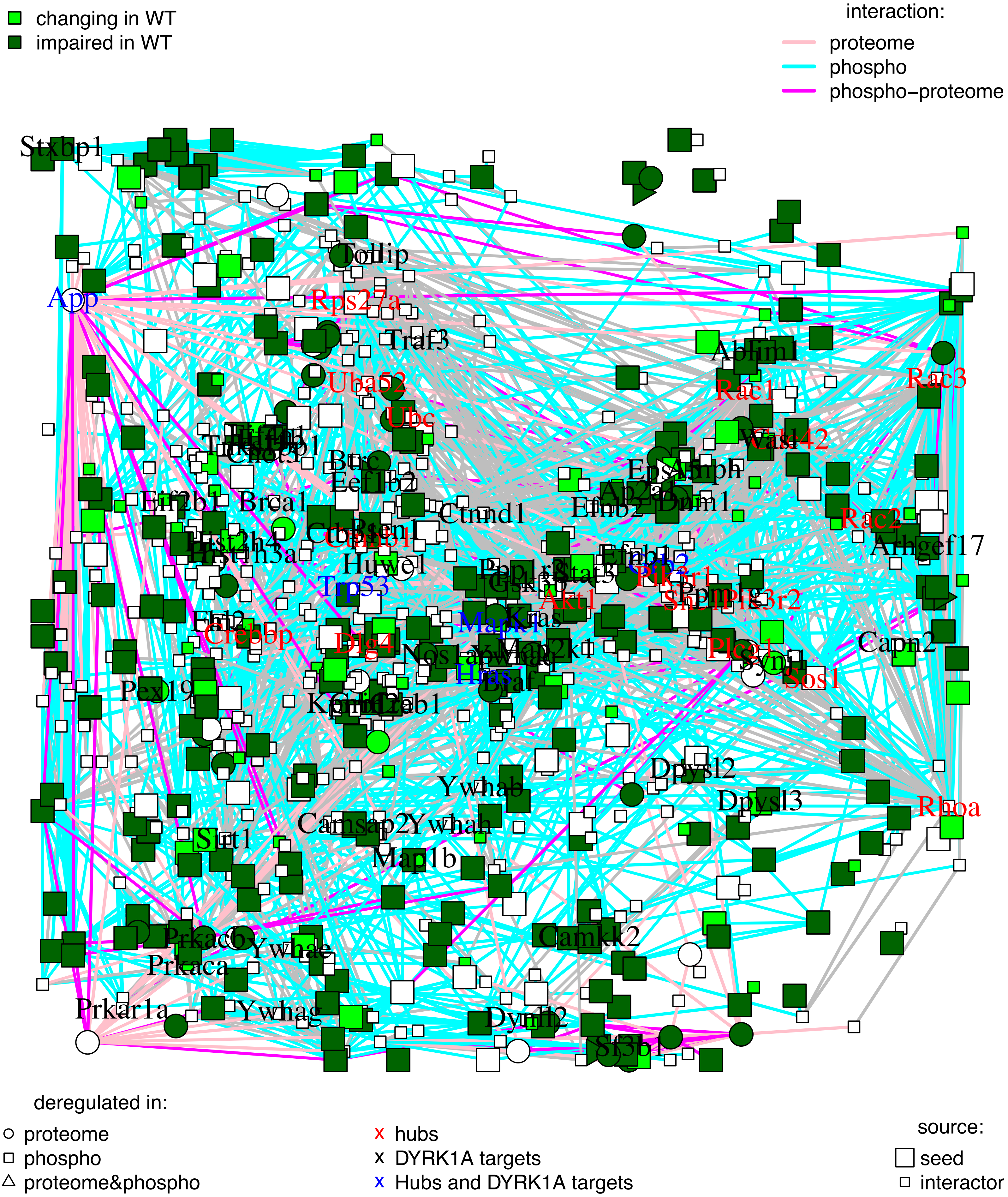

Supplementary Figure 7: Protein-protein interaction network of proteins changing in Ts65Dn mice compared to wild type mice. Graph where the nodes correspond to proteins changing in Ts65Dn (seeds) and its direct interactors, and edges correspond to known protein-protein interactions. Seed proteins are visualized as bigger nodes, circles correspond to protein changing their abundances, squares to proteins changing their phosphorylation, triangles to proteins changing both their abundance and their phosphorylation levels. Protein changing upon treatment in wild type mice are colored in green, dark green if their levels are significantly changing in the direction of TS untreated levels. Interactions involving a protein changing its abundance are represented in pink, if involving a protein chancing its phosphorylation level in cyan, if involving protein changing both abundance and phosphorylation in purple. The thickness of the edge is proportional to the interaction score. Names of DYRK1A interactors and hubs are printed out. Black: DYRK1A interactors. Red: hubs. Blue: both DYRK1A interactors and hubs.

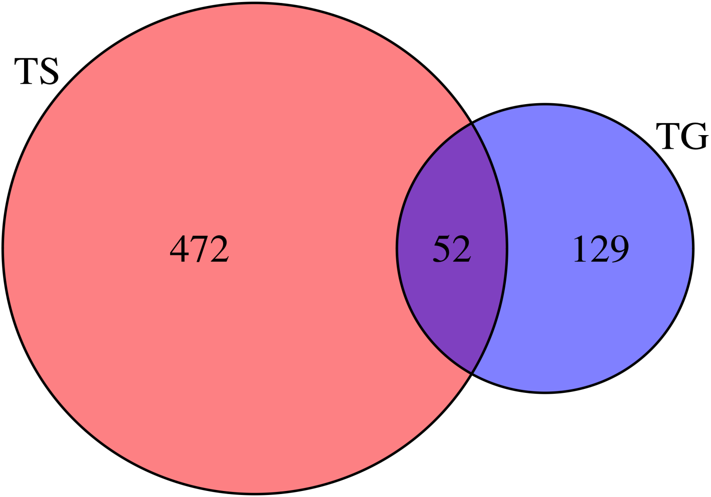

Supplemental Figure 8: Comparison of Ts65Dn mice and TgDyrk1A mice. VennDiagram showing the overlap of list of proteins rescued by green tea, EE, or both treatments in Ts65Dn mice (TS, in red) with those rescued in Dyrk1A transgenic mice (TG, in blue).
